# Implementation of Adenovirus-Mediated Pulmonary Expression of Human ACE2 in HLA Transgenic Mice Enables Establishment of a COVID-19 Murine Model for Assessment of Immune Responses to SARS-CoV-2 Infection

**DOI:** 10.3390/pathogens10080940

**Published:** 2021-07-26

**Authors:** Theodor Chitlaru, Erez Bar-Haim, Liat Bar-On, Shahar Rotem, Hila Cohen, Uri Elia, David Gur, Moshe Aftalion, Ron Alkalay, Efi Makdasi, Yentl Evgy, Reut Falach, Ma’ayan Israeli, Adi Bercovich-Kinori, Hagit Achdout, Yfat Yahalom-Ronen, Ronit Rosenfeld, Ofer Cohen

**Affiliations:** 1 Department of Biochemistry and Molecular Genetics, Israel Institute for Biological Research, P.O. Box 19, Ness-Ziona 74100, Israel; erezb@iibr.gov.il (E.B.-H.); liatb@iibr.gov.il (L.B.-O.); shaharr@iibr.gov.il (S.R.); hilac@iibr.gov.il (H.C.); urie@iibr.gov.il (U.E.); gurd@iibr.gov.il (D.G.); moshea@iibr.gov.il (M.A.); rona@iibr.gov.il (R.A.); yentle@iibr.gov.il (Y.E.); reutf@iibr.gov.il (R.F.); maayani@iibr.gov.il (M.I.); adiki@iibr.gov.il (A.B.-K.); ronitr@iibr.gov.il (R.R.); 2 Department of Infectious Diseases, Israel Institute for Biological Research, P.O. Box 19, Ness-Ziona 74100, Israel; Efim@iibr.gov.il (E.M.); hagita@iibr.gov.il (H.A.); yfatyr@iibr.gov.il (Y.Y.-R.)

**Keywords:** adenovirus vector, human ACE2, SARS-CoV-2, COVID-19, HLA transgenic mice

## Abstract

HLA transgenic mice are instrumental for evaluation of human-specific immune responses to viral infection. Mice do not develop COVID-19 upon infection with SARS-CoV-2 due to the strict tropism of the virus to the human ACE2 receptor. The aim of the current study was the implementation of an adenovirus-mediated infection protocol for human ACE2 expression in HLA transgenic mice. Transient pulmonary expression of the human ACE2 receptor in these mice results in their sensitisation to SARS-CoV-2 infection, consequently providing a valuable animal model for COVID-19. Infection results in a transient loss in body weight starting 3 days post-infection, reaching 20–30% loss of weight at day 7 and full recovery at days 11–13 post-infection. The evolution of the disease revealed high reproducibility and very low variability among individual mice. The method was implemented in two different strains of HLA immunized mice. Infected animals developed strong protective humoral and cellular immune responses specific to the viral spike-protein, strictly depending on the adenovirus-mediated human ACE2 expression. Convalescent animals were protected against a subsequent re-infection with SARS-CoV-2, demonstrating that the model may be applied for assessment of efficacy of anti-viral immune responses.

## 1. Introduction

SARS-CoV-2 virus, the etiological pathogen responsible for the current COVID-19 pandemic, recognises and binds the human angiotensin converting enzyme II receptor (hACE2) on target host cells in the course of infection [1,2]. ACE2 receptor engagement resulting in cellular internalization is essential for the virus pathogenicity, and abrogation of the receptor recognition prevents the development of the disease. Accordingly, the viral spike protein responsible for the contact with the hACE2 receptor represents the target for prophylactic pre- and post-exposure therapies (for review, see [3]). Notably, the human tropism of the SARS-CoV-2 virus is attributed at a large extent to the preferential specificity of the spike protein to its cognate hACE2 receptor and lower affinity to analogous ACE2 receptors from related phylogenetic hosts [4]. The essentiality of the presence of the ACE2 receptor for the development of the disease was demonstrated by the observation that mice, which normally are not susceptible to SARS-CoV-infection, can be sensitized by heterologous expression of the hACE-2 receptor. Mice expressing the human ACE2 receptor represent an essential animal model for study of the COVID-19 disease and, most importantly, for the evaluation of urgently needed therapeutic approaches. As of today, two general approaches have been reported for hACE2-mediated sensitization of mice to SARS-CoV-2 infection: first, transgenic mice expressing a hACE2 receptor allele, originally developed for the study of infection and pathogenesis of SARS-CoV and repurposed for the study of SARS-CoV-2 [5]. While these mice provide a valuable tool for the study of SARS-CoV-2, they are limited in their versatility, being restricted to a single genetic background. Furthermore, these mice express hACE2 in tissues other than those in which is naturally expressed and often exhibit non-physiological hypersensitivity to SARS-CoV-2 [6,7]. The second approach is in vivo expression of hACE2 cloned in engineered viral vehicles such as adenovirus (Ad) or adeno-associated virus (AAV). The adenovirus-mediated expression of the recombinant hACE2 receptor (mediated by hybrid viruses abbreviated here as Ad-hACE2) emerged as an excellent system for generating a transient state of SARS-CoV-2 susceptibility, which can be implemented for recapitulating the major manifestations of COVID-19 (evaluated and quantified by weight loss and viral load) in transduced mice. Most importantly, the system enabled the evaluation of SARS-CoV-2 countermeasures [8,9].

Here, we report the implementation of the Ad-mediated hACE2 expression in transgenic mice expressing the human leukocyte antigen (HLA)-class 1 (HLA mice). HLA mice, owing to the humanized nature of their major histocompatibility complex, are frequently used as immunological models of human viral disease and vaccine development [10]. Their lymphocytes are known to recapitulate human-like presentation of antigens resulting in generation of HLA-restricted cytotoxic T lymphocytes (CTL) epitopes. Accordingly, the transgene enables physiological peptide selection by human class 1 molecules in murine antigen presenting cells, and an appropriate CD8+ T-cell repertoire containing T-cell receptors (TCRs). Of note, the HLA-A2 allele (represented by the transgene expressed in the humanized mice employed in this study; see below) is highly prevalent within the human population. Accordingly, specific epitopes generated by T lymphocytes expressing this allele are an important target for dissecting the T-cell response associated with human infections and malignancies [11].

The protocol was implemented in two strains of HLA transgenic mice. The first one is a C57BL/6-derived commercially available strain of transgenic mice, exhibiting the HLA-A2 transgene in the background of a resident murine histocompatibility complex (MHC-1). The second expresses the HLA-A2 transgene in the background of MHC-null (“knock-out”) in which the expression of the entire resident murine MHC was abrogated. We show that intra-nasal (IN) infection of the mice with Ad-hACE2 enables the expression of hACE2 in the lungs of the animals resulting in their susceptibility to SARS-CoV-2 infection, which is manifested by transient weight loss. The development of disease was strictly dependent on the expression of hACE2. Infected animals elicited a strong humoral and cellular immune response against the viral spike protein, which conferred full protection against a subsequent re-challenge with the virus.

## 2. Results

### 2.1. Transduction of HEK-293 Cells and Intranasal Infection of C57BL/6 Mice with an Adenovirus-hACE2 Vector Result in Expression of hACE2

**Figure 1:**
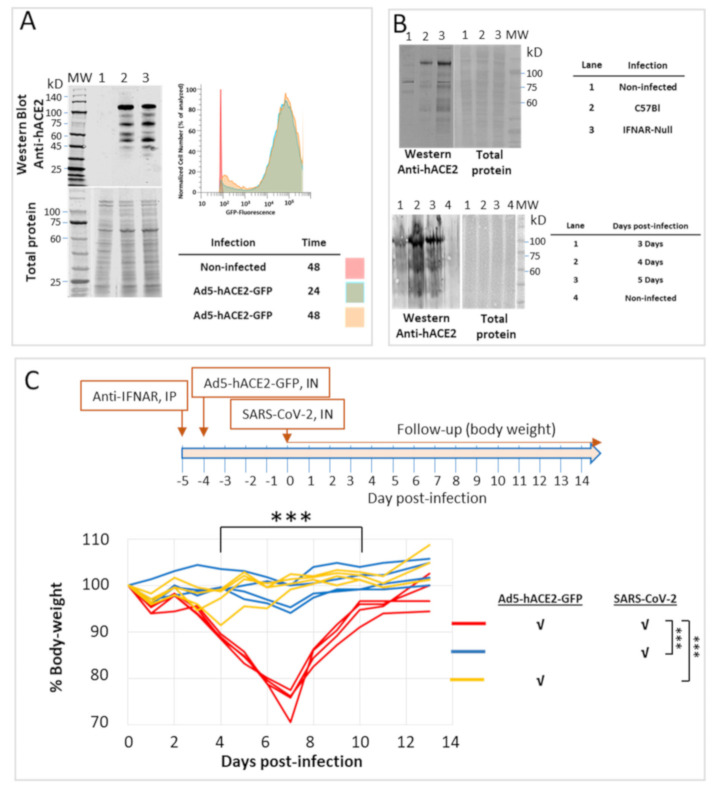
Expression of hACE2 and GFP using the Ad5-hACE2-GFP vector. (**A**) Left panel: Western blot analyses of HEK-293 cells transduced with the Ad5-hACE2-GFP vector, collected at the indicated time points, using anti-ACE2 antibodies. Right panel: FACS analysis of cells for detection of GFP expression. Identity of lanes: MW-Molecular Weight markers; 1-Non-infected cells; 2-infected cells harvested 24 h post-infection; 3-infected cells harvested 48 h post-infection. (**B**) Optimization of ACE2 expression. Upper panel: Western blot analysis using anti-hACE2 antibodies of lungs extracted from mice of non-infected C57BL/6 (lane 1), or Ad5-ACE2-GFP-infected (IN) C57BL/6 (lane 2) or IFNAR-Null mice (lane 3), as indicated in the legend at the right. Lungs were extracted 5 days post infection. Lower panel: Western blot analysis using anti-hACE2 antibodies of lungs extracted at the indicated times from C57BL/6 mice IN infected with the Ad5-hACE2-GFP vector and pre-administered with anti-IFNAR antibodies (by IP injection). (**C**) Upper scheme indicates the various steps in the infection of C57BL/6 mice. As indicated, mice (*n* = 4) were transduced with the Ad5-hACE2-GFP vector (8 × 10^8^ PFU/mouse) 24 h after administration of anti-IFNAR antibodies (2 mg/mouse, IP); 4 days later mice were infected IN with 10^6^ CFU SARS-CoV-2 and weighed daily. The body weight curves represent percentage of the initial weight (of the day of SARS-CoV-2 administration, which is considered 100%). The body weight of each individual mouse is presented separately. The legend on the right explains the three experimental groups. Statistics: the body weight change was evaluated by a two-way ANOVA with Bonferroni’s post-test: *** *p* < 0.001.

An engineered commercial adenovirus 5 (Ad5) was employed as a vector for hACE2 expression in the current study. The virus was modified by replacement of the viral early genes with a hACE2 gene under the control of the CMV promoter and includes a GFP reporter gene carrying its own CMV promoter. Prior to in vivo experiments, the vector referred to as Ad5-hACE2-GFP was confirmed to enable the expression of hACE2 and GFP in HEK-293 cells. Indeed, 24 h post-transduction of cells at a multiplicity of infection (MOI) higher than 10, significant amounts of hACE2 could be detected by Western blot analyses using specific anti-hACE2 (Figure 1A, left panel). Similarly, FACS analysis confirmed expression of the GFP reporter gene (Figure 1A, right panel).

Expression of hACE2 was then interrogated in the lungs of infected C57BL mice. Abrogation of the interferon-alpha/beta receptor (IFNAR) system was reported to increase the efficiency of the adenovirus-mediated expression of hACE2 and subsequent susceptibility to SARS-CoV-2 infection [8,9]. This aspect was confirmed by comparative Western blot analyses of the amount of hACE2 in the lungs of C57BL/6 and IFNAR-1 null mice. In line with previous observations, the absence of a functional IFNAR system resulted in an augmented amount of hACE2 expression (Figure 1B, upper panel). Accordingly, all subsequent experiments described in this report included administration of anti-IFNAR monoclonal antibodies by IP injection, 24 h prior to the IN infection with the Ad5-hACE2 vector, as previously recommended [8]. The issue of IFNAR-blocking contribution was not further addressed and we cannot rule out the possibility that it is not essential for implementation of the model. Furthermore, in cases in which evaluation of various treatments may be affected by IFNAR blockage, apparently it may be omitted for subsequent quantification of infection by qPCR [8].

The time of maximal hACE2 expression following IN infection of mice with the Ad5-hACE2-GFP was determined to be 4 days post-transduction of mice with Ad5-hACE2-GFP (Figure 1B, lower panel). Optimal expression of hACE2 receptor occurred following IN administration of 8 × 10^8^ PFU. Lower doses resulted in considerably less hACE2 expression and higher doses were not attempted (not shown). Of note, differences may exist between different laboratories with respect to the quantification of Ad5-hACE2 viral stocks. In the present study, the concentrations of the stocks were considered those specified by the manufacturer of the recombinant Ad5-hACE2 preparations (see “Methods”).

The data depicted in Figure 1C documents the follow-up of IN infection of C57BL/6 mice with SARS-CoV-2. As indicated in the scheme (Figure 1C), animals were administered with the Adeno-hACE2-GFP vector 4 days prior to infection. Control animals were administered either with SARS-CoV-2 only, or with the Ad-hACE2-GFP viral vehicle only. A decrease in body weight, starting on day 3, was recorded for all mice in the experimental group composed of hACE2-expressing animals infected with SARS-CoV-2, but not for those in the control groups. The maximal decrease in body weight was reached on day 7 and the animals regained their original weight within the following 6 days. Very low variability was determined between the animals. These results are in line with those previously reported for C57BL/6 mice transduced with an Adenovirus derived hACE2 expression vector [9].

### 2.2. SARS-CoV-2 Infection of Ad5-hACE2-Transduced HHD and HLA-A2.1 Humanized Transgenic Mice

**Figure 2:**
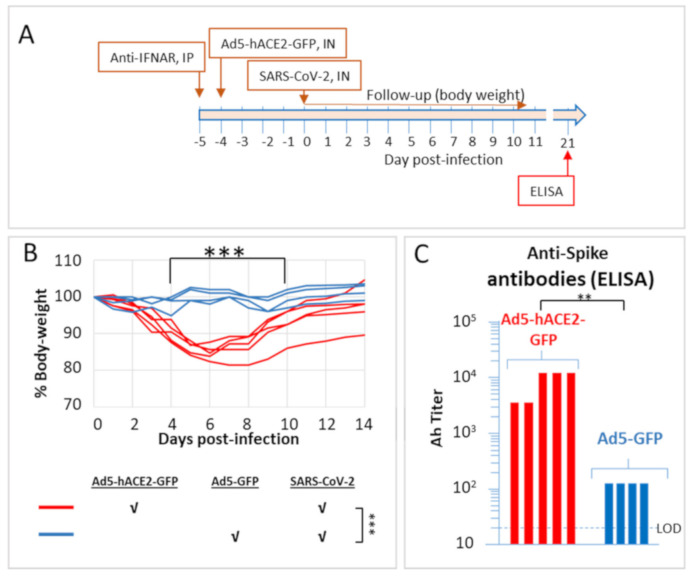
Weight change of HHD transgenic mice transduced with Ad5-hACE2-GFP and infected with SARS-CoV-2 according to the scheme in (**A**). (**B**) As indicated in the lower legend, mice (*n* ≥ 4) were transduced with the Ad5-hACE2-GFP vector or with an Ad5 vector expressing only the reporter GFP gene (8 × 10^8^ PFU/mouse) 24 h after administration of anti-IFNAR antibodies (2 mg/mouse, IP). Four days later mice were infected IN with 10^6^ CFU SARS-CoV-2, and weighed daily. The body weight curves represent the percentage of the initial weight (on the day of SARS-CoV-2 administration). The body weight of each individual mouse is presented separately. Statistics: the body weight change was evaluated by a two-way ANOVA with Bonferroni’s post-test: *** *p* < 0.001. (**C**) Blood was collected from the animals and analysed by ELISA for detection of anti-spike antibodies twenty-one days after infection. The ELISA limit of detection (LOD) is indicated by a dotted line. Naive murine sera exhibited ELISA titres lower than the LOD (not shown). Statistics: mean values of the two experimental groups were analysed by a Student t-test, ** *p* < 0.01.

The sensitization of mice to infection with SARS-CoV-2 by Ad5-hACE2 expression was further demonstrated in two strains of HLA transgenic mice. Both strains are useful for infectious disease research, vaccine development and testing, safety and immunogenicity testing, as well as research directed towards study of various immune disorders. Furthermore, they permit identification of epitopes restricted to the human HLA specificity [11,12]. The first strain consisted of HHD-2, which express a human HLA class-I molecule instead of the murine MHC complex. The data described in Figure 2 demonstrates, as in the case of C57BL/6 mice, that hACE2 expression results in the development of disease which is manifested by a transient decrease in body weight. SARS-CoV-2 infection of control mice transduced with an engineered Adenovirus expressing the GFP reporter gene only, did not reveal any detectable loss of weight after SARS-CoV-2 infection. Titres of anti-spike protein antibodies were determined by ELISA in sera collected from the mice twenty-one days after the infection. All convalescent mice exhibited high titres of anti-spike antibodies, considerably superior to those determined in mice exposed to SARS-CoV-2 without previous transduction with the Ad5-hACE2 vector (Figure 2C). This observation is in line with the concept that the disease elicits a significant humoral response, much stronger than in the case when exposure to the virus did not result in the development of the disease (such as in the case of animals that do not express hACE2).

**Figure 3:**
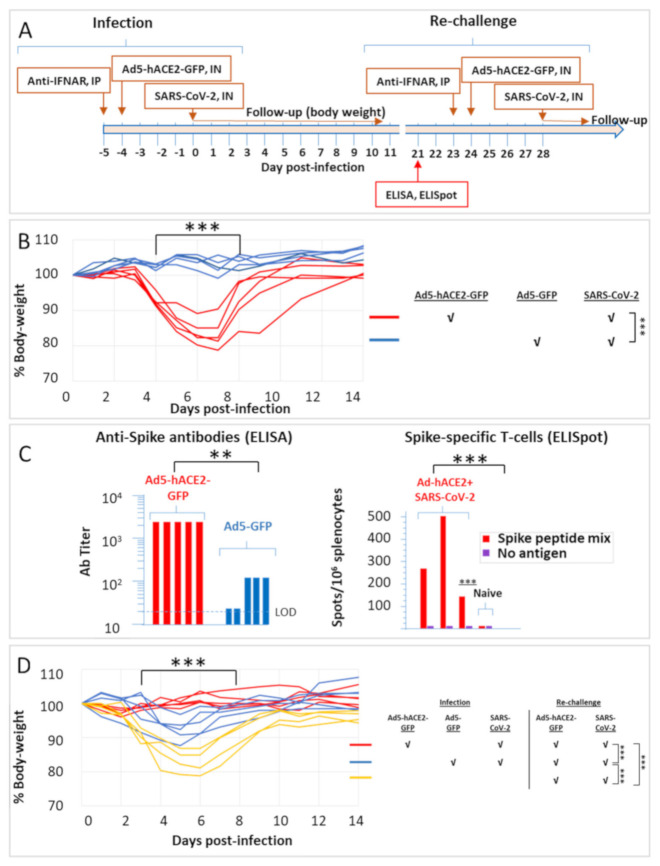
Weight change of HLA transgenic mice transduced with Ad5-hACE2-GFP and infected with SARS-CoV-2 according to the scheme in (**A**). (**B**) As indicated in the lower legend, mice (*n* = 5) were transduced with the Ad5-hACE2-GFP vector or with an Ad5 vector expressing only the reporter GFP gene (8 × 10^8^ PFU/mouse) 24 h after administration of anti-IFNAR antibodies (2 mg/mouse, IP). Mice were infected IN with 10^6^ CFU SARS-CoV-2, four days later, and weighed daily. The body weight curves represent percentage of the initial weight (on the day of SARS-CoV-2 administration). The body weight of each individual mouse is presented separately. Statistics: the body weight change was evaluated by a two-way ANOVA with Bonferroni’s post-test: *** *p* < 0.001. (**C**) Blood was collected from the animals twenty-one days after infection and analysed by ELISA for detection of anti-spike antibodies or by ELISPOT for quantification of anti-spike specific T-cells. The ELISA limit of detection (LOD) is indicated by a dotted line. Naive murine sera exhibited ELISA titres lower than the LOD (not shown). Statistics: mean values of the two experimental groups were analysed by a Student t-test, ** *p* < 0.01, *** *p* < 0.001. (**D**) All animals were re-challenged with bSARS-CoV-2 twenty-four days after infection, according to the scheme in panel A. In parallel, four naive animals were challenged with the virus, as indicated in the legend on the right. Statistics: the body weight change was evaluated by a two-way ANOVA with Bonferroni’s post-test: *** *p* < 0.001.

Ad5-hACE2 transduction was subsequently implemented for COVID-19 modelling in HLA-A2.1 transgenic mice. These mice carry a chimeric human HLA-murine MHC molecule [13]. As in the case of the HHD mice, infection resulted consistently into a significant reversible decrease in body weight only of hACE2 expressing mice, starting on day 3 post-infection, reaching maximal low level on day 6 and exhibiting full recovery on day 14 (Figure 3B). At twenty-one days post-infection, blood samples collected from the convalescent mice exhibited high titres of anti-spike antibodies (Figure 3C), significantly higher than infected animals that did not express hACE2 (and therefore did not exhibit any decrease in body weight). Three animals were sacrificed for quantification of the spike-specific T-cells in their spleens, by ELISPOT assays (Figure 3C). The data clearly establishes that exposure to SARS-CoV-2 of hACE2-expressing mice results in the mounting of a significant cellular immune response, specific for the viral spike protein.

At twenty-four days following infection, all mice were transduced with Ad5-hACE2 and re-challenged with SARS-CoV-2 (Figure 3D, see also scheme in Figure 3A). While naive animals revealed the typical reversible weight loss, all convalescent animals did not exhibit any sign of disease following the re-challenge. Animals that did not express hACE2 (transduced with the control Ad5-GFP vector, blue curves in Figure 3B) but were exposed to SARS-CoV-2 in the first infection, exhibited a minor yet consistent decrease in body weight following the challenge (which included transduction with Ad5-hACE2-GFP and SARS-CoV-2, blue curves in Figure 3D). This observation precludes the possibility that the protective immune response exhibited by convalescent animals originates from an inadvertent anti-Adenovirus immune response elicited by the transduction with the ACE2-expressing vector prior to the first exposure. Taken together, the data indicate a strong correlation between the titre of anti-spike antibodies and the resilience to infection, strongly supporting the notion that the COVID-19 model generated in the HLA-humanized mice by Adenovirus-mediated expression of hACE2, may serve for the evaluation of protective immunity against SARS-CoV-2 infection.

In conclusion, the current report demonstrates the applicability of Adenovirus transduction as a means for expression of hACE2 in HLA-humanized mice and their consequent sensitization to SARS-CoV-2 infection. Owing to its versatility, this approach will contribute to assessment and characterization of specific human immune responses as well as interrogation of the efficacy of various therapeutic strategies.

## 3. Materials and Methods

### 3.1. Cells and Virus Strains

Human epithelial HEK-293 (ATCC^®^ CRL-1573TM) and Vero E6 (ATCC^®^ CRL-1586TM) cells, obtained from the American Type Culture Collection (Manassas, VA, USA) were used and maintained in Dulbecco’s modified Eagle’s medium (DMEM) supplemented with 10% foetal bovine serum (FBS), non-essential amino acids (NEAA), 2 mM L-Glutamine, 100 Units/mL Penicillin, 0.1 mg/mL Streptomycin and 12.5 Units/mL Nystatin (P/S/N) (Biological Industries, Israel). Cells were cultured at 37 °C, 5% CO_2_ at 95% air atmosphere. SARS-CoV-2 (GISAID accession EPI_ISL_406862) was kindly provided by Bundeswehr Institute of Microbiology, Munich, Germany. Stocks were prepared by infection of Vero E6 cells for two days. When viral cytopathic effect (CPE) was observed, media were collected, clarified by centrifugation, aliquoted and stored at −80 °C. Titer of stock was determined by plaque assay using Vero E6 cells. Ad-GFP-hACE2 (code ADV-200183) and Ad-CMV-GFP (code 1060-HT) were purchased from Vector Biosystems INC (Malvern, PA, USA), aliquoted and stored at −80 °C until use. Handling and working with SARS-CoV-2 was conducted in a BL3 facility in accordance with the biosafety guidelines of the IIBR.

### 3.2. Transduction and Harvest of of Cells for Analysis

HEK293 cells were seeded 24 h prior to infection in 6-well microtiter plates at approx. 30% confluency (10^5^ cells/well). For transduction, the cells were washed with PBS and covered with 0.5 mL DMEM devoid of FCS containing the adeno-virus at various MOIs, for 3 h. The cells were washed with PBS and the media was replaced with fresh DMEM. At 24 to 48 h post transduction, the cells were washed and scraped into PBS, centrifuged and re-suspended either in Laemli SDS-PAGE buffer supplemented with 100 mM (final) DTT (Dithithreitol, Bio-Rad) or FACS flow-buffer (PBS supplemented with 1% FBS and 0.05% sodium azide).

### 3.3. Experimental Animals

All animal experiments were approved by the IIBR committee for animal research (protocol M-33-20). The experimental animals were handled according to the National Research Council 1996 Guide for the Care and Use of Laboratory Animals and regulations of the IIBR Animal Use Committee. Female C57BL6 mice (6–8 weeks old) were obtained from Jackson (Bar Harbor, Main, USA). HLA-A2.1 transgenic mice (CB6F1-Tg [HLA-A*0201/H2-Kb] A*0201) were purchased from Taconic (Germantown, NY, USA). HHD-2 mice are β2-microglobulin (β2m)−/−, Db−/− and express chimeric HLA class-I molecule composed of the human β2-microglobulin, HLA-A*0201 α-1 and α-2 domains and the mouse α-3 domain of H-2Db. HHD-2 mice were a kind gift from Prof. Lea Eisenbach, Department of Immunology, Weizmann Institute of Science, Israel. HHD mice were bred in the IIBR animal facility. The expression of the transgene was confirmed in all transgenic animals. The mice were allowed free access to water and rodent diet (Envigo, Israel).

### 3.4. Infection of Animals

Mice were injected intraperitoneally (IP) with 2 mg anti IFNAR monoclonal antibodies in 0.5 mL, 24 h prior to infection with the Ad5-derived viruses. For intra-nasal (IN) administration of adenovirus, anaesthesia was applied by IP injection of Ketamine/xylazine (50 and 5 mg, respectively, per kg body weight); 25 μL of Ad5-ACE2 were administered to each nostril, and four days later, mice were administered with 10^6^ PFU (plaque forming units) SARS-CoV-2 virus under anaesthesia. Statistical analysis of the animal experimentation data is indicated in the respective figure legends.

### 3.5. Antibodies

Anti-mouse IFNAR-1 (interferon-α/β receptor) purified in-vivo GOLD antibodies (code I-401-100) were purchased from LEINCO Technologies, Inc (St. Louis, MI, USA). Anti-ACE2 antibodies (HD14AP2302-B) were from Sino Biologicals US Inc. (Wayne, PA, USA). Secondary antibodies for Western blot analyses were IRDye^®^ 800CW conjugated goat anti-rabbit. Primary and secondary antibodies were used at 1:500 and 1:20,000 dilutions respectively.

### 3.6. Collection of Lungs for Evaluation of ACE2 Expression by SDS-PAGE and Western Blot Analysis

Mice lungs were dissociated in GentleMACS C-tubes (Miltenyi Biotec, Germany) and resuspended in 2 mL PBS. Forty microliters were re-suspended in Laemli SDS-PAGE buffer (Bio-Rad) supplemented with 100 mM (final) DTT for Western blot analysis. SDS-PAGE was carried out on 4–12% NuPage Bis-Tris gels (Invitrogen) using Precision Plus Molecular weight markers (Bio-Rad). Western blots were generated using the Nitrocellulose Western iBlot Gel transfer Semi-dry system (Invitrogen). The nitrocellulose membranes were blocked in LiCor blocking buffer for 1 h at room temperature, and probed with primary antibody overnight at 4 °C. The membranes were washed three times for 10 min in PBST (PBS containing 0.05% Tween), probed with secondary antibody for 1 h at room temperature and washed twice. The blots were scanned using the LiCor laser-based image detection method. Excelband Molecular weight markers were from SMOBIO Technology (Taiwan).

### 3.7. ELISA and ELISPOT

A standard direct ELISA protocol was applied essentially as described [6]. Microtiter plates were coated using 2 μg/mL of recombinant SARS-CoV-2 spike, AP-conjugated anti-mouse IgG (Jackson ImmunoResearch, USA), was used as secondary antibody. Detection was performed using PNPP substrate (Sigma, Israel) according to manufacturer directions. ELISPOT assays were carried out essentially as described [14]. Briefly, spleens were dissociated in GentleMACS C-tubes (Miltenyi Biotec, Bergisch Gladbach, Germany), passed through 70-micron mesh, and splenocytes were separated on lympholyte-M media according to the manufacture protocol. Single-cell suspensions were seeded into 96-well ELISPOT plates (Cellular Technology Limited, Cleveland, OH) 4 × 10^5^ cells/well, with strict adherence to the manufacturer’s instructions. For antigen stimulation, a commercial pool of 315 peptides and 15 amino-acids long were used (PM-WCPV-S, JPT Peptide Technologies GmbH, Berlin, Germany). The frequency of IFNγ-secreting cells was quantified with ImmunoSpot S6 Ultimate reader and analysed with the ImmunoSpot software (Cellular Technology Limited, Cleveland, OH, USA).

### 3.8. Flow Cytometry

In total, 293 transfected cells were harvested and washed with flow buffer (PBS with 1% FBS and 0.05% sodium azide). Cells were re-suspended in flow buffer and collected on a Fortea flow cytometer (BD Bioscience, San Jose, CA, USA). Data were analysed using the FlowJo software (Tree Star, version 10).

## Data Availability

All data pertaining to the study described in the manuscript is described in the report.
